# Evaluation of Cystic Echinococcosis Cases in Terms of Sociodemographic, Clinical and Hospitalization Features in Karaman Province, Turkey

**Published:** 2019-12

**Authors:** Mehmet Fatih AYDIN, Emre ADIGÜZEL

**Affiliations:** Faculty of Health Sciences, University of Karamanoglu Mehmetbey, Karaman, Turkey

**Keywords:** Cystic echinococcosis, Hospital, Hospitalization, Turkey

## Abstract

**Background:**

We aimed to investigate the cystic echinococcosis (CE) epidemiology in Karaman Province from 2010 to 2017 using data from the provincial state hospital.

**Methods:**

Overall, 482 cases were determined from Karaman State Hospital, Karaman Province, Turkey from 2010 to 2017. Records were investigated in terms of year, gender, age, cyst location, rural and urban households and duration of hospital stay.

**Results:**

The CE incidence was 22.40 per 100 000 people after final diagnosis with focused abdominal ultrasound. Totally, 482 people including 166 (34.4%) male and 316 (65.6%) female were with CE. The mean age of patients was 50.3±17.48 yr. More CE cases were reported in the age group of 51–60 yr than in the other age groups. CE was mostly seen in liver (470, 97.5%). Moreover, 28.4% of patients were resided in rural areas. The observed frequencies of demographic features of female gender, infected liver, residence in provincial centers, referral to general surgery policlinic and no hospitalization were significantly higher than expected frequencies (*P*<0.05). Sixty (12.4%) patients with CE had been hospitalized. There was a statistically significant positive relationship between age of hospitalized patients and duration of hospitalization (*P*<0.05). Of the patients 73.2% were admitted to general surgery.

**Conclusion:**

CE is of great importance to public health considerations in the Karaman Province and we advocate the implementation of eradication programs to decrease the CE cases number.

## Introduction

Cystic echinococcosis (CE) is a zoonotic parasitic disease caused by the larval form (metacestode) of *Echinococcus granulosus*, lives as adult forms in the small intestines of dogs and other carnivores, and characterized by cysts in internal organs of sheep, goats, cattle and also humans (1). The disease is transmitted by the consumption of infected green vegetables and water. In addition, soil and dog feathers can also mediate transmission of the disease (2).

Cystic echinococcosis remains an important public health problem throughout the world, despite innovations in diagnostic, therapeutic and control programs.

Some studies using serological methods and hospital records have been conducted to determine the CE epidemiology in various provinces of Turkey (3–8). Examination of hospital records provides practical, easy and important information to determine CE epidemiology in humans. Since cases are examined by these retrospective and descriptive studies, it is also possible to determine the potential risk factors for the disease. To the best of our knowledge, there is no adequate information for CE epidemiology in Karaman Province of Turkey.

This study aimed to investigate the epidemiology, clinical and hospitalization features for CE in Karaman Province from 2010 to 2017 using data from Karaman State Hospital.

## Methods

### Study Area and Population

Karaman Province, located at 37.11 latitude and 33.15 longitudes, is 1033 m above sea level and has a surface area of 8869 km^2^. Karaman can show temperate climate characteristics towards the south while generally having continental climate characteristics. It has an annual average rainfall of 336.3 kg/m^2^. Agriculture, animal husbandry and related industrial sector activities have an important place in the Karaman economy. Karaman Province had a population of 246 672 in 2017 and nearly half of the people lived in rural areas (9). The province has four hospitals, two of which are state and the others are private. This study was carried out in Karaman State Hospital that is the most equipped and the most applied hospital in the province.

### Data Collection and Statistical Analysis

For the study, the permission obtained from General Secretariat of Karaman Province Public Hospitals Union with the letter dated 02.03.2017 and numbered 99–667.

Patients, diagnosed between 01.01.2010 and 31.12.2017, were evaluated for year of diagnosis, gender, age, organ involvement, hospitalization period, and residence place. The universe of the study was patients diagnosed with CE at Karaman State Hospital between 01.01.2010 and 31.12.2017, and the sample was determined as the whole of the universe.

SPSS 16 statistical package program (ver. 16.0, Chicago, IL, USA) was used for statistical evaluation. Data is expressed as number (n), percentage (%), mean (X), standard deviation (SD), median (M) and interquartile range (IQR). Levene's and Kolmogorov-Smirnov tests were used to assess homogeneity and normality respectively. For comparison of expected and observed frequencies, binomial test was used in two groups and one-sample chi-square test was used in more than two groups. Because data is not normally distributed, Mann Whitney U and Kruskal Wallis tests were used for comparison of groups according to group number. Spearman’s rank correlation analysis method was used to compare continuous variables. *P*<0.05 was considered to show a statistically significant result.

## Results

There were 482 CE cases diagnosed with focused abdominal ultrasound at Karaman State Hospital. The percentage of male patients was 34.4% (n=166) and that of female patients was 65.6% (n=316). The mean and median ages were 50.3±17.48 and 51±24 yr, respectively. In the vast majority of patients (97.5%) the involved organ was the liver. Generally (71.6%) living place was provincial centers. The most frequent outpatient clinic was the general surgeon with a percentage of 73.2%. In addition, 12.4% of the patients were clinically followed at the hospital. Descriptive characteristics of female gender, liver as an involved organ, residence in provincial centers, applying to general surgery clinic and no hospitalization were found to have a higher observed frequency than expected frequency at the significant level (*P*<0.05) (Table 1).

**Table 1:** General characteristics of patients

***Features***	***n***	***%***	**P*-value***	***Test***
**Gender**				
Male	166	34.4	0.000^*^	Binomial test
Female	316	65.6		
**Organ involvement**				
Lung	12	2.5	0.000^*^	Binomial test
Liver	470	97.5		
**Residence**				
City center	345	71.6	0.000^*^	One sample chi-square test *X*^*2*^=319.498
District center	55	11.4		
Small town-village	82	17.0		
**City**				
Karaman	429	89.0	0.000^*^	One sample chi-square test *X*^*2*^=1057.469
Mersin	36	7.5		
Konya	12	2.5		
Other	5	1.0		
**Polyclinic**				
General surgery	353	73.2	0.000^*^	One sample chi-square test *X*^*2*^=1913.046
Internal diseases	42	8.7		
Gastroenterology	36	7.5		
Thoracic surgery	17	3.5		
Pediatric surgery	13	2.7		
İnfectious disease	8	1.7		
Emergency internal diseases	8	1.7		
Pediatry	3	0.6		
Chest diseases	2	0.4		
**Hospitalization**				
Yes	60	12.4	0.000^*^	Binomial test
No	422	87.6		

* *P*<0.05

**Fig. 1:**
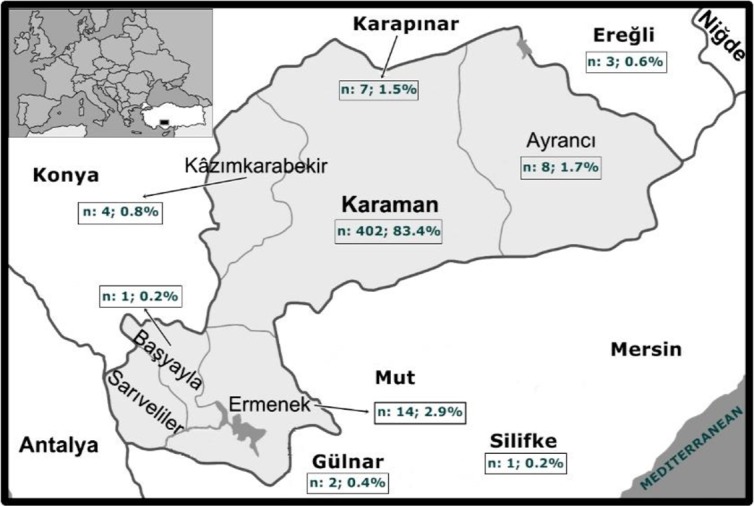
Distribution of cases of cystic echinococcosis in Karaman province borders and surrounding provinces

The majority of patients (89.0%) reside in Karaman provincial borders (Fig. 1). The disease was diagnosed mostly between 51–60 yr (n:111, 23.0% of all cases) and at least between 0–20 yr (n:26, 5.4% of all cases).

The largest number of cases were seen in the year 2013 (n: 103, 21.4% of all cases) and at least in 2010 (n: 10, 2.1% of all cases).

Disease diagnosis frequency is given according to years in Table 2. The diagnosis frequency was the highest in 2013 (39.09/100000) and was the lowest in 2010 (3.44/100000).

**Table 2:** CE diagnostic frequency according to years in Karaman Province

***Year***	***Population***	***Number of diagnoses***	***Diagnostic frequency (per 100 000)***
2010	232 633	8	3.44
2011	234 005	37	15.81
2012	235 424	22	9.34
2013	237 939	93	39.09
2014	240 362	71	29.54
2015	242 196	53	21.88
2016	245 610	67	27.28
2017	246 672	78	31.62
Incidence of CE between 2010–2017 is 22.40/100 000			

The mean hospitalization time of patients was 0.9±2.79 d between 2010 and 2017 with no statistically significant difference by gender (*P*<0.05). Length of stay in hospital was significantly lower in the 41–50 age group than the 0–20 and 31–40 age groups (*P*<0.05). In addition, length of stay in hospitals in the 51–60 age group was significantly lower than the 0–20 age group (*P*<0.05). The hospitalization times in 2010 was significantly higher than the other years (*P*<0.05). Moreover, hospitalization times of patients reside provincial center was significantly lower than those of patients reside district centers (*P*<0.05).

The mean and median hospitalization times of hospitalized patients (n = 60) were 7.3±3.99 and 7±4 d respectively. Hospitalization rates were 16.3% in males and 10.4% in females. According to age group, the highest hospitalization rate was found in the 0–20 yr age group (30.8%) and the lowest hospitalization rate in the 41–50 age group (4.1%). The rate of hospitalization in 2010 was 60.0%, on the other hand this rate decreased to 4.8% in 2017. In addition, 9.0% of the hospitalized patients residing in the provincial center; and 23.2% of them living in towns and villages (Table 3).

**Table 3:** The mean (*X̄*), standard deviation (SD), median (M) and interquartile range (IQR) values of duration of hospitalization and number of hospitalized patients according to features

Features^*^	***Duration of hospitalization (day)***		**P-*value***	***Test***	***Number of hospitalized patients***		P-value^***^	***Test***
	***X̄ ±SD***	***M±IQR***			***n***	***%***		
**Gender**								
Male (n=166)	1.2±3.24	0±0	0.069	Mann	27	16.3	0.519	Binomial test
Female (n=316)	0.8±2.52	0±0		Whitney U Z= −1.821	33	10.4			
**Age group**								
0–20 yr (n=26)	1.9±3.30^a^	0±4	0.000^**^	Kruskal-Wallis *X^2^*= 26.336	8	30.8	0.264	One sample chi-square test *X^2^*equals;7.667
21–30 yr (n=38)	1.3±2.69^a,b^	0±1			9	23.7		
31–40 yr (n=67)	1.6±3.18^a,c^	0±0			15	22.4		
41–50 yr (n=98)	0.3±1.33^b^	0±0			4	4.1		
51–60 yr (n=111)	0.5±2.32^b,c^	0±0			8	7.2		
61–70 yr (n=81)	0.9±2.62^a,b^	0±0			9	11.1		
71 yr and over (n=61)	1.3±4.29^a,b^	0±0			7	11.5		
**Years**								
2010 (n=10)	5.6±7.37^a^	4.5±8	0.000^**^	Kruskal-Wallis *X^2^*equals; 42.304	6	60.0	0.310	One sample chi-square test *X^2^*equals;8.267
2011 (n=41)	1.4±2.91^b,c^	0±0			9	22.0		
2012 (n=27)	2.7±4.76^b^	0±6			8	29.6		
2013 (n=103)	1.0±2.82^c,d^	0±0			12	11.7		
2014 (n=86)	0.6±1.75^c,d^	0±0			9	10.5		
2015 (n=58)	0.7±1.84^c,d^	0±0			9	15.5		
2016 (n=73)	0.4±1.90^d^	0±0			3	4.1		
2017 (n=84)	0.4±2.31^d^	0±0			4	4.8		
**Residence**								
City center (n=345)	0.6±2.33^a^	0±0	0.001^**^	Kruskal-Wallis *X^2^*equals;	31	9.0	0.004^**^	One sample chi-square test *X^2^*equals;11.100
District center (n=55)	1.3±2.91^b^	0±0			10	18.2		
Small town-village (n=82)	1.8±4.07^b^	0±0		14.184	19	23.2		

* Percentages were calculated separately for each group (according to the number n),

** *P*<0.05

a,b,c,d *P*<0.05 for groups of different letters and *P*>0.05 for groups of the same letter,

*** *P*-values were calculated for hospitalized patients (n=60)

There was a statistically significant positive correlation between age of hospitalized patients and duration of hospitalization (r=0.292; *P*<0.05; Spearman’s rank correlation).

## Discussion

Cystic echinococcosis, an important public health problem, caused by the larval form of *E. granulosus*, characterized by cysts in internal organs of intermediate hosts, also is a chronical disease and causes sudden deaths.

CE is endemic in many countries and Turkey because of ineffective eradication programs, inadequate antiparasitic medication in stray dogs, uncontrolled animal slaughter, misapplications due to lack of information in humans (10).

The incidence of CE in the region was 22.40/100000 with this study. Overall incidence of CE in Turkey for 2001–2005 years was 6.30/100000 and this was 5.23/100000 for Karaman Province in the same period (11). In the same study, the highest rate was determined in Kırklareli with 23.28/100000 while the lowest rate was determined in the cities of Black Sea Region (0–2.81/100000). Therefore, CE has reached very serious dimensions in Karaman Province. According to the report published in 1999 by FAO CE incidence was 3.5/100000 in Greece, 1.92/100000 in Italy, 2.2/100 000 in Portugal and 0.78/100000 in Spain (12).

In similar studies conducted in Middle East countries, CE incidence was slightly higher than Europe countries. The incidence of CE was 1.5/100000 in Iran (13), 2.1/100000 in Palestine (14), 2.3/100000 in Jordan (15) and 4.5/100000 in Iraq (16).

All of the CE cases determined by this study did not apply to the hospital primarily with complaints regarding CE. Cysts detection during coexisting diseases (data not presented) are being investigated provide high success in CE diagnosis. A great number of stray dog population in the region and imperfect knowledge and improper practices of the community may cause the increasing the incidence of CE in Karaman Province when compared to other provinces. In another study conducted by our team, the butchers in Karaman Province had not enough information and have improper practices on CE and thus could contribute to spread of the disease (17). Cystic echinococcosis is a chronic illness. The agent needs a long time to develop into a cyst diagnosed by imaging techniques after entering the body (18).

In this study, 72.8% of CE cases were seen after 40 yr of age and most cases were detected in 51–60 age group (23.0%). According to many researchers most CE cases were reported after 40 yr of age in human (4,13,19,20). Furthermore, 77% (14) of patients diagnosed with CE in Palestine and 81.69% in Iraq were under 50 yr of age (16). High prevalence of food-borne infections and discrepancy of socio-economic, cultural, geographical and climatic factors in these geographies may be important factors for “CE cases in Palestine and Iraq were mostly seen before 50 yr of age”. Although the disease is seen more frequently in later ages, it is important to carry out control and awareness-raising studies from childhood due to the fact that the agent enters the body at an early age.

Although CE is frequently seen in both genders, some factors may increase the incidence of the disease especially in women (16). In many studies, the disease was detected at higher rates in women. (14,16). Women's agricultural and livestock activities can provide more encounter with the pathogen. Another reason why women have more illness is that they may benefit from health services more than men because of other health problems. Cystic echinococcosis can be seen in many internal organs, especially in the liver and lungs in intermediate hosts. In this study, 97.5% (470/482) of the CE patients had liver involvement. In similar studies performed, liver involvement was detected at high rates (21–25).

Cystic echinococcosis can be seen more frequently in areas where agriculture and animal husbandry are intensively performed, where effective control measures are not taken adequately and where there is insufficient awareness of risk factors (26,27).

Although Karaman Province center is considered as an urban area, it can be considered as a rural settlement area because the majority of its population is in agriculture and animal husbandry activities. In this study, 71.6% of the CE cases were located in provincial centers and 28.4% were living in rural areas such as districts, towns, and villages. Different studies also support the findings of this study (4,19,28).

73.2% of CE patients applied to general surgery, 8.7% of internal medicine and 7.5% applied to gastroenterology polyclinic in this study. General surgery (43.8%), infectious diseases (21.9%) and gastroenterology (21.9%) were the most frequently applied polyclinics when CE cases between 2009 and 2013 in Çorum were investigated (19). Our results were similar to the results of this study. In Kars, the most frequently applied policlinic was chest surgery with 45.8% followed by general surgery with 41.1% and pediatric surgery with 13.1% (20). High rate diagnosis of CE in the chest surgery clinic in Kars may be due to high incidence of pulmonary involvement.

Cystic echinococcosis is a disease with high treatment cost due to labor loss and economic losses. Surgical intervention and hospitalization increase the cost of treatment. When the CE cases between 2010 and 2017 were examined in Karaman, 60 patients were hospitalized for a total of 439 d. In Turkey, between 2001 and 2005, 14789 CE-diagnosed patients were hospitalized for 149464 d in total (11).

In this study, when the hospitalization duration of cases was compared according to age groups, hospitalization times of individuals between 0–40 yr were found to be slightly higher than those of other age groups. There was also a decrease in the rate of hospitalization with the increase in age and the progress of years. When the CE cases between 1998 and 2016 were scanned in Brazil, the high hospitalization time, particularly in the 20–40 age group (29), is consistent with the results of this study. In this study, there was a decrease in the rate of hospitalization as well as hospitalization duration with the increase of age groups and with the progress of years.

When hospitalized CE records in Spain between 1997 and 2012 were examined, hospitalization rates were decreasing according to years and the frequency of hospitalization was significantly higher at later ages (*P*<0.05) (30). Cystic echinococcosis can be diagnosed as primary or it can accompany other diseases. Young people diagnosed only with CE were hospitalized at a higher rate. There are also other chronic diseases in elderly patients diagnosed with CE.

## Conclusion

CE is an important public health problem with the frequency of 22.40/100000. There was an increase over the previous years, it was more common in women than in men and the most affected organ was the liver. Furthermore, CE can be seen at the same frequency with the villages and towns in the rural provincial centers where the people more engaged in agriculture and livestock breeding. Cystic echinococcosis is thought to cause a significant burden on the economy and labor force as a result of hospitalization. It is necessary to reduce the number of CE cases by applying education programs and effective control programs.

## Ethical considerations

Ethical issues (Including plagiarism, informed consent, misconduct, data fabrication and/or falsification, double publication and/or submission, redundancy, etc.) have been completely observed by the authors.
